# COVID-19 among LGBTQ+ individuals living with HIV/AIDS: psycho-social challenges and care options

**DOI:** 10.3934/publichealth.2021023

**Published:** 2021-03-23

**Authors:** Aditi Tomar, Mandy N Spadine, Taylor Graves-Boswell, Lisa T Wigfall

**Affiliations:** 1 Department of Health Education and Kinesiology, College of Education and Human Development, Texas A&M University, USA; 2 MD Anderson Cancer Center, USA

**Keywords:** HIV/AIDS, LGBTQ+, psychosocial, COVID-19

## Abstract

Prolonged social isolation during the COVID-19 lockdown has adversely impacted the mental, social, and physical wellbeing of the global populace. Coping with mental and physical stressors amidst the global lockdown is especially strenuous for the Lesbian, Gay, Bisexual, Transgender, and more (LGBT+) community, who are frequently subjected to social stigma and minority stress. Systematic stigma and discrimination place LGBT+ individuals at higher risk for deleterious behaviors, such as substance abuse (e.g., injection drug use, smoking, alcohol) and risky sexual practices (e.g., anal/vaginal/oral sex). Maladaptive coping behaviors consequently increase the chances of HIV/AIDS risk among LGBT+ individuals, compared to heterosexual individuals. LGBT+ individuals Living with HIV/AIDS perpetually face higher rates of unemployment, income disparity, and intimate partner violence. Prolonged home confinement, and impaired accessibility to healthcare, legal, and criminal justice services during lockdown may deplete the quality of life of LGBT+ individuals Living with HIV/AIDS. Therefore, it is critical that multidisciplinary service providers, including health professionals, employers, social services providers, educational institutions and community organizations, move toward online service delivery, so that homebound HIV-positive LGBT+ individuals are secured with a wide range of care options. Non-judgemental, tele-counseling may bridge the gap to mental health services. Community clinics catering to HIV-positive and/or LGBT+ clients may consider precociously supplying essential amenities, such as Preexposure (PrEP)/postexposure prophylaxis (PEP), condoms, emergency contraception, and sterile needles. Lastly, efforts directed at the sustenance of at-risk/HIV-positive LGBT+ health should persevere, even after the pandemic.

The COVID-19 pandemic has led to a state of legally-enforced and self-imposed social distancing, resulting in persistent social isolation [1]. Prolonged social isolation has adversely impacted the mental, social, sexual, and physical wellbeing of the global populace [2]. Coping with mental and physical stressors amidst the global lockdown is especially strenuous for the Lesbian, Gay, Bisexual, Transgender, Queer and more (LGBTQ+) community, who are frequently subjected to social stigma and minority stress [3].

LGBTQ+ individuals face markedly elevated psycho-social stress, including systematic stigmatization, and have higher rates of HIV-risk behaviors (alcohol, substance abuse, unsafe sexual behaviors) [4],[5]. As a result, they bear a disproportionate burden of Human Immunodeficiency Virus/Acquired Immune Deficiency Syndrome (HIV/AIDS) and HIV/AIDS-related co-infections [6]. Particularly, Men-having-sex with men have a 22 times greater likelihood of becoming infected with HIV compared to their heterosexual counterparts [7]. In the same vein, transgender women have a 12-fold susceptibility to acquiring HIV infection, compared to the general population [7]. Collectively, the high prevalence of risky maladaptive practices, psychological stressors and HIV/AIDS-associated perpetual immunocompromised state (low CD4 count), may put LGBTQ+ Living with HIV/AIDS at a much higher risk of contracting COVID-19 [8].

The prolonged lockdown has led to an alarming rise in physical and psycho-social burdens among LGBTQ+ individuals Living with HIV/AIDS [9],[10]. A recent survey conducted in July 2020 by The Trevor Project reported that one in four LGBTQ+ youth, were unable to access mental health care, three-fourths LGBTQ+ participants indicated increased loneliness and depression, and over half (53%) indicated experiencing symptoms of depression during the worldwide, COVID-19 lockdown [11]. Another survey, conducted by the UNAIDS, the LGBTQ+ Foundation, Johns Hopkins Bloomberg School of Public Health and, other global institutions shows that close to a quarter (21%) global LGBTQ+ populace, living with HIV experienced “restricted access” to Antiretroviral therapy refills [12].

The COVID-19 economic crisis has caused massive truncation in workforce, around the globe [13]. The rampantly occurring layoffs and job loss during the COVID-19 crisis have exacerbated the pre-existing, sub-optimal socio-economic burden among this stigmatized group [12]. Furthermore, the dual-minority identity of LGBT+ individuals living HIV (as HIV+ and LGBT+), increases their likelihood of experiencing poverty, unemployment, homelessness, and food insecurity [14]. Ongoing challenges such as lower health insurance, income, and employment rates, which are inordinately prevalent among LGBTQ+ living with HIV/AIDS, have worsened during the lockdown [15],[16]. The UNAIDS survey results referenced previously, depict that as of May 2020, 13% LGBTQ+ survey respondents were unemployed, and close to a quarter (21%) were worried about losing jobs in the future [12].

In addition to social discrimination and stigmatization, LGBTQ+ individuals bear an unfortunate burden of higher intimate partner violence (IPV) rates, relative to their heterosexual, cisgender counterparts [17]. Reportedly, most abusers threaten to divulge the victim's sexual/gender identity to homophobic/transphobic friends, family, and/or employers, as a means to usurp power and control [18]. Threats related to identity disclosure situation may be far more serious among individuals wanting to conceal their dual HIV/AIDS-LGBTQ+ status. The repercussions of IPV may be life-threatening in situations where perpetrators resort to violence, and afflict physical injury to their partners [19]. Home confinement during the COVID-19 pandemic hinders victims from seeking medical and/or legal assistance from criminal justice authorities, support groups and/or supportive friends and family [9]. Apart from preventing HIV-positive LGBTQ+ from seeking adequate support for mental health, the current stay-at-home orders have hindered access to antiretroviral treatment and HIV medical attention [20].

The predisposition to HIV-risk behaviors (particularly unprotected sex) among LGBT+ individuals has exacerbated during the ongoing pandemic. The UNAIDS survey shows that 13% LGBTI respondents reported reduced ability to negotiate for safer sex, 1% have turned to sex work, and 2% were continuing to sell sex; during the pandemic [21]. The continual engagement in unsafe sexual practices, despite orders of strict isolation put LGBTQ+ at a much higher risk for acquiring both HIV and coronavirus.

At this juncture, we must acknowledge the impact of the structural inequities in worsening the health and socio-economic burden among HIV-positive LGBT+ individuals within social minority communities. In the United Sates, structural inequities are known to widen intergroup disparities in healthcare access, and promote stigma, stress, and political marginalization of these communities [22]. As a result, racial/ethnic minority groups, especially Afro-Americans and Hispanics inordinately suffer from adverse health conditions. It must be noted that Black and Hispanic/Latino MSM are at particularly high risk for HIV, compared with MSM from other communities; and structural racism during the current pandemic increases their susceptibility to COVID-19 [22],[23].

The ongoing pandemic has grievously impacted both the mental and physical well-being of HIV-positive LGBTQ+ individuals [23]. Limited accessibility to support services amplifies risk to COVID-19, as well as other chronic illnesses [24]. To add, LGBTQ+ people living with HIV bear a high threat to discrimination in health care, and thereby refrain from adequately seeking medical support and mental health assistance [25]. At this point, it is critical that multidisciplinary LGBTQ+ service providers, including health professionals, employers, social services providers, educational institutions and community organizations, move toward online service delivery [24],[26]. Particularly, both mental health providers, and medical professionals need to utilize a tailored-approach, to suit the needs of LGBTQ+ individuals living with HIV/AIDS.

The effective utilization of telemedicine services in expanding HIV care across LGBTQ+ communities has been demonstrated previously [27]. LGBTQ+ clients seeking telemedicine-led HIV care appreciate the continuity in health care, especially during these unprecedented, “social distancing” times [28]. In addition, the benefits of tele-counselling in mitigating the mental health ramifications of COVID-19 have been widely discussed [29]. While delivering mental health services to LGBTQ+ individuals living with HIV/AIDS, adopting a non-judgmental, friendly responses are critical to fostering trust and confidence [30]. At the same time, introducing provider-friendly policies will ease service delivery and safeguard providers from incurring financial losses [31],[32]. For example, many insurance companies are allowing therapists to bill for online therapy during the pandemic. Additionally, some states in the U.S. have permitted leniency in licensure expiration dates and/or requesting therapists with expired licenses return to the field [33],[34].

Medical efforts targeted towards HIV-positive/at-risk LGBTQ+ individuals must aim towards improving accessibility to routine HIV care and testing, providing screening services for HIV co-infections and COVID-19, and alleviating discrimination against LGBTQ+/HIV-positive individuals in a cost-friendly healthcare setting. Community clinics catering to the HIV-positive and/or LGBTQ+ clients may consider precociously supplying essential amenities, such as Preexposure (PrEP)/postexposure prophylaxis (PEP), condoms, emergency contraception, sterile needles, and lubricants [35]–[38]. These clinics may also serve as hubs for connecting clients to peer support services and domestic violence shelters and refuges.

Since the World Health Organization's release of the HIV self-testing guidelines in 2016, HIV self-tests have gained global acceptability among a wide-range of at-risk communities [39]. At-home HIV testing effectuates early diagnosis, and is particularly relevant during the ongoing pandemic. It offers fast results within the ease and comfort of one's home; promotes social-distancing and restricts community exposure to COVID-19 [39]. Other recommendations include, implementing flexible health services such as mobile clinics in low socio-economic areas and supplying ART refills lasting at least six months [40]. Restrictive measures imposed during the ongoing pandemic have exacerbated the pre-existing health disparities among at-risk/HIV-positive LGBT+ individuals. Clinical and social efforts towards uplifting at-risk/HIV-positive LGBTQ+ individuals, should persevere even after the pandemic; with specialized attention directed towards high-risk minority groups.
